# Factors Influencing Sustainable Development Literacy among Engineering Undergraduates in China: Based on the College Impact Model

**DOI:** 10.3390/ijerph20021249

**Published:** 2023-01-10

**Authors:** Shuyu Qi, Danning Huang, Qiutong Ma, Mi Zhou

**Affiliations:** School of Higher Education, Faculty of Humanities and Social Science, The Capital Research and Development Center for Engineering Education, Beijing University of Technology, Beijing 100124, China

**Keywords:** sustainable development literacy, institutional support, student engagement, engineering education

## Abstract

Achieving carbon neutrality is a major strategy to combat climate change and achieve sustainable development. Training engineering undergraduates with sustainable development literacy is an important way to achieve this goal in the field of higher education. Based on the college impact model, this research surveyed 1070 engineering undergraduates in Chinese universities to explore the influence of institutional support on undergraduates’ sustainable development literacy, and the empirical analysis was conducted using Structural Equation Modeling. The results showed that institutional support positively and significantly influenced the sustainability development literacy of engineering undergraduates, and student engagement had a mediating role in the relationship between institutional support and sustainability development literacy. Relevant recommendations for cultivating engineering undergraduates with high-level sustainability development literacy are proposed here.

## 1. Introduction

The most important driver of current global climate change is the emission of carbon dioxide and other greenhouse gases due to human activities, which is one of the most pressing challenges in the world’s environmental problems today. The global carbon neutrality target set by the Paris Agreement has set the direction of development for countries to reduce carbon emissions. As the second largest economy and educational power in the world, China has always been a strong advocate and active practitioner of the concept of sustainable development, and pledged to achieve a carbon peak by 2030 and carbon neutrality by 2060. Sustainable development (SD) has been a key strategy to combat climate change and achieve carbon neutrality. The Brundtland report defines sustainable development as “the ability to meet the needs of present without compromising the ability of the future generations to meet their own needs” [1]. To safeguard the environment, social well-being, and economic well-being for both present and future generations, next generation must be educated to become literate in sustainability [2]. Education for sustainable development (ESD) is the process of developing students’ knowledge and attitudes that motivate behavioral change in favor of sustainability and the environment [3,4]. This definition outlines the learning outcomes (e.g., sustainable development literacy) that students need to acquire in order to perform according to demands of sustainable development in the world of work and in society [5].

As a beacon of social progress and development, higher education institutions (HEIs) should take the lead in exploring the direction of human progress and provide the world with the knowledge base and value guidance of sustainable development. Sustainable development first reached prominence in higher education with the Talloires Declaration in 1990 [6]. This declaration discussed the role of higher education in sustainable development and the need for education for sustainable development. “Universities educate most of the people who develop and manage society’s institutions. For this reason, universities bear profound responsibilities to increase the awareness, knowledge, technologies, and tools to create an environmentally sustainable future” [7]. At the same time, the declaration stipulated three requirements for sustainable development literacy, namely raising awareness on environmentally sustainable development, educating for environmentally responsible citizenship, and fostering environmental literacy for all. The Talloires Declaration provides guidelines for higher education institutions to develop sustainable development literacy in their students. Higher education institutions are important places to educate the next generation of professionals [8] by teaching curricula that connect classroom-learned knowledge with life and the world, as well as extracurricular activities that provide experience in community projects and voluntary activities; cultivate students’ knowledge, attitudes, behaviors, and social responsibility about sustainable development [9,10,11]; and promote students to become citizens with sustainable development literacy.

Due to the nature of engineering disciplines, engineering education is at the forefront of responding to the sustainable development of society. As the fourth industrial revolution continues to advance, systematic changes in industrial production patterns and human lifestyles are occurring, resolving the conflicts between humans and nature and promoting the sustainable development of industry and society. At present, China has built the world’s largest higher engineering education system [12], with the number of engineering undergraduates enrolled accounting for 35% of the total scale of engineering education worldwide [13]. Thus, whether higher engineering education can cultivate high-quality talents with sustainable development literacy is directly related to whether the country and the world can effectively respond to environmental changes and achieve the goal of carbon neutrality. Education for sustainable development focuses on the sustainability of individual students, and for the field of engineering education, this requires higher education organizations or engineering accreditation commissions to construct training objectives and learning outcomes that promote the sustainable development of engineering undergraduates. In September 2015, UNESCO adopted the 2030 Agenda for Sustainable Development, which sets out 17 sustainable development goals, 8 of which are closely related to engineering education for sustainable development (e.g., Climate Action, Life below Water). Based on the United Nations sustainable development goals, the International Engineering Alliance has updated Graduate Attributes and Professional Competencies from the Washington Agreement [14] and the Accreditation Board for Engineering and Technology has updated Student Outcomes [15] to ensure that engineering undergraduates have sustainable knowledge, attitudes and behaviors.

Today, learning outcomes for sustainable development in higher education are still a point of contention. In the higher education literature on this topic, the learning objectives of sustainability education have many labels, such as competencies [16] or literacy [17,18]. However, the main consensus in the literature is that sustainable education should involve cognitive, socio-emotional, and behavioral domains of students [19,20]. The cognitive domain comprises knowledge and thinking skills necessary to better understand sustainable development and the challenges in achieving it. The socio-emotional domain includes social skills that enable learners to collaborate, negotiate, and communicate to promote sustainable development, as well as self-reflection skills, values, attitudes and motivations that enable learners to develop themselves. The behavioral domain describes action competencies [21]. In this study, sustainable development literacy is selected to study the sustainable development learning achievements of engineering undergraduates. Sustainable development literacy for engineering undergraduates refers to the mastery of the basics of sustainable development and the frontiers of engineering expertise to better understand the current reform goals and realistic challenges in global sustainable development, the awareness and values of sustainable development to be able to self-reflect and make ethical judgments when facing problems in sustainable development, and the ability to consider and assess the impact on sustainable development when solving engineering problems.

Reviewing previous research, some scholars have begun to examine the teaching and learning of sustainability in higher education by studying pedagogy [22] or learning outcomes [23,24] or by integrating sustainability themes into disciplinary curricula [25]. However, there are still some limitations in the current studies. In terms of research object, there is a lack of investigation and research for fourth-year engineering undergraduates. As preparatory engineers, fourth-year engineering undergraduates are the core human resources for advancing sustainable development of enterprises and society. Therefore, there is an urgent need to test the effectiveness of higher education institutions in cultivating sustainable development learning outcomes of engineering undergraduates and guaranteeing their ability to serve sustainable development in their future careers. In terms of research content, previous studies have focused on the impact of individual educational factors (e.g., teaching [26], pedagogy [27], community engagement [28], and campus practices [29]) on students’ sustainable development outcomes and lack a systematic and comprehensive empirical framework. The college impact model has been shown to be a more comprehensive framework for explaining student outcomes, but only a few individual studies [30,31] have introduced the college impact model to examine the causal relationship between college experiences and engineering undergraduates’ sustainability learning outcomes, but have not introduced student engagement as a mediating variable. Pascarella [32] argues that the quality of student effort and engagement are the most important factors influencing student development, that the college environment can only be truly effective through the mediation of student engagement, and that it is futile to discuss the influential role of the institution without student engagement. At the same time, little research has focused on the moderating role of students’ individual characteristics (e.g., gender) in the relationship between the college experience of engineering undergraduates and sustainable learning outcomes.

To address these limitations of existing research, this study constructs a model of factors influencing engineering undergraduates’ sustainable development literacy based on the theory of the college impact model and explores three key questions to be addressed: (1) to examine the effects of three aspects of institutional support (i.e., supportive environment, sustainability curriculum, instructional practice) on engineering undergraduates’ sustainable development literacy; (2) to examine the mediating role of two aspects of student engagement (i.e., classroom experience, extracurricular involvement) in the relationship between institutional support and engineering undergraduates’ sustainable development literacy; and (3) to explore the moderating role of gender in the relationship between institutional support, student engagement and sustainable development literacy of engineering undergraduates. The focus of this study is to conduct a survey for 1194 engineering undergraduates to assess the effectiveness of higher education institutions in developing learning outcomes and cultivation pathways for the sustainable development of engineering undergraduates. It also proposes policy recommendations on how to improve the sustainable development literacy of engineering undergraduates and provides a reference theoretical basis and research to promote the sustainable development of engineering education and the achievement of China’s carbon neutrality goals.

## 2. Theoretical Basis and Research Hypothesis

### 2.1. College Impact Model

The college impact model focuses on the influence of colleges and universities on individual student development, and is widely used to analyze the impact of the college environment on student development and the ability to improve while enrolled in college. Research on the impact of college on students links student development to the college environment, individual student characteristics, and learning behaviors, focusing on the “interactive effects” of student engagement and the college environment [33,34]. The college impact model has been developed since the 1960s, based on the input–environment–output (I-E-O) model originally proposed by Astin [35], and several individual–environment interaction models have been developed, among which the typical and representative theoretical model is Pascarella’s general model for assessing change [36]. Pascarella’s model states that structural/organizational characteristics of institutions, student background/precollege traits, institutional environment, interactions with agents of socialization, and quality of student effort all have direct or indirect impact on student learning outcomes.

Sustainability education for engineering undergraduates is comprehensive and transformative, involving learning outcomes, instructional practices and institutional environments. The purpose of this study is to comprehensively discuss the relationship between institutional factors and the sustainable development literacy of engineering undergraduates. Pascarella’s general model for assessing change has a high degree of fit with the content of this study; thus, the study used this theoretical model as the basis for establishing initial model. By combing through the relevant literature over the years, Pascarella et al. [34] found that the impact of college on students depends on two main aspects: the level of effort and engagement of university students, and the various supports provided by institutions to encourage and engage students. This study explicitly investigates the impact of three aspects of institutional support (i.e., supportive environment, sustainability curriculum, instructional practice) and two aspects of student engagement (i.e., classroom experience, extracurricular involvement) on the sustainable development literacy of undergraduate engineering undergraduates. Institutional support directly influences the sustainable development literacy of engineering undergraduates, and indirectly influences the sustainable development literacy of students through student engagement. Within the framework of the college impact model, students’ background characteristics (e.g., gender) may influence the differential relationship between engineering undergraduates’ college experiences and their learning outcomes. Therefore, this study used gender as a moderating factor.

### 2.2. Institutional Support and Sustainable Development Literacy

In the college impact model, institutional support is a key factor in student learning outcomes and has a direct impact on student competency development. Morly [37] has found that the comprehensive support of institution for students is an important factor affecting the systematic changes in students. Tinto [38] proposed a model of institutional action for student success that further focuses on institutional support from the campus environment, proposes institutional support factors, and explores the impact of institutional support on students. Higher education could demonstrate its practical contribution to sustainable development through its campus environment. The campus environment is both a physical entity but also a representation and mediator of values and culture, playing a potential role in shaping and influencing student attitudes, behaviors, and learning [39]. The physical infrastructure of the campus and the campus culture have an impact on the daily experience of faculty, staff, and students.

Formal curriculum is an important way to promote student learning or learning sustainability. Research has shown that students can be systematically provided with sustainability knowledge through the integration of sustainability topics into the curriculum. Horvath et al. [40] found a significant difference in sustainability knowledge between respondents who reported taking zero courses and those who took three or more courses. Cotton et al. further demonstrated this connection from studying energy literacy in higher education. They found that 50% of student respondents cited formal education as their main source of information contributing to their understanding of energy issues [41]. Tang [42] found that a sustainable development course provided in Curtin University Malaysia yielded positive effects on students’ sustainability beliefs, attitudes, and behavioral intentions. Prior research has shown that sustainability learning increases when students are exposed to this topic in higher education classrooms. In fact, taking just one sustainability course has been recognized to increase students’ pro-sustainability behaviors [43].

Promoting students learning about sustainability requires consideration not only of the level of student exposure to sustainability-related content, but also of the instructional practices employed to promote learning of sustainability-related content. The pedagogical approaches represent the general character or guiding principles of designing learning processes in sustainability education. Cotton and Winter proposed a number of instructional practices to promote students’ sustainable development competences, such as group discussions, stimulus activities (watching a video or looking at photos, poems, or newspaper extracts to initiate reflection or discussion), debates, critical incidents (students are given an example and asked what they would do, what they could do, and what they should do), case studies, and problem-based learning [4]. However, there is limited empirical research on how instructional practices affect engineering undergraduates’ sustainability. The correlation analyses showed a relationship between the contribution to sustainability and the strength of competences, and between the strength of competences and the strength of pedagogical approaches [44]. Yusof [45] demonstrated that problem-based learning (PBL) can instill professional skills and promote sustainable development in first-year engineering undergraduates. Jollands and Parthasarathy [46] discovered that project-based learning, as a promising approach for promoting sustainability education, significantly improved the understanding of sustainability among chemical engineering undergraduates. Some experiments have confirmed that students’ cognitive learning outcomes are better when they increase their use of community-oriented and constructive learning approaches. In other words, multi-method experiential active learning education improves sustainable cognitive learning [47].

According to the theory of the college impact model and the existing empirical research on sustainable development literacy, this study categorizes supportive environment, sustainability curriculum, and instructional practice as institutional support factors, and proposes the following hypothesis:

**H1.**  *Institutional support has a significant positive effect on sustainable development literacy*.

### 2.3. Mediating Role of Student Engagement

The college impact model states that institutional support not only affects student learning outcomes directly, but also indirectly by influencing student engagement [32]. Astin defined student involvement as the amount of physical and psychological energy that a student devotes to the academic experience [48]. Following the development of subsequent studies, Kuh [49] introduced the concept of student engagement and argued that student engagement includes both the extent to which students participate in educationally effective activities as well as their perceptions of facets of the institutional environment that support their learning and development. Carini, Kuh, and Klein [50] examined student engagement as associated with students’ academic learning outcomes and found that many measures of student engagement, including student engagement, were positively linked with such learning outcomes. In this study, student engagement is defined as the time and energy students invest in sustainable learning during effective educational activities inside and outside the classroom.

Although the classroom is the primary site of education for sustainable development implementation in higher education institutions, less research has focused on student engagement experiences in the classroom. Education for sustainable development often focuses too narrowly on instilling knowledge about sustainable development, but rather on appropriate pedagogy that truly engages learners in learning about sustainable development. Classroom experiences where students are actively reflecting and learning are essential to learning about sustainability. Howell used the flipped classroom survey to conclude that students’ knowledge of sustainability in the course was enhanced through reflective and active learning. Over 90% of students agreed that in-class active learning exercises made the classes more engaging and the material more memorable than usual [51].

In addition to formal classroom learning, the learning experiences students have outside of the classroom are important to their overall learning. The term “extracurricular” is typically used to denote learning activities outside of a formal curriculum [52]. The distinguishing features of informal curriculum activities are that they are largely student-directed, voluntary, open to all, and non-credit-bearing. Students are involved in extracurricular activities including volunteering, internships, clubs and societies, and events. In the context of sustainability learning, extracurricular experiences are valuable because they move students beyond awareness and toward engaging with environmentalism in their everyday lives [53]. Extracurricular interventions have a high utility to help advance education for sustainability in higher education [54]. Lipscombe et al. [52] surveyed 140 United Kingdom HEIs to explore the types of sustainability extra-curricular interventions being used; an important discovery from this survey was over half of the survey respondents felt these extra-curricular interventions helped to advance education for sustainable development.

Based on the college impact model and available empirical evidence, this study suggest that institutional support may first influence students’ engagement in sustainability learning and then increase their sustainability literacy. Therefore, this study proposes the following hypothesis:

**H2.**  *Student engagement mediates the relationship between institutional support and sustainable development literacy*.

### 2.4. The Moderating Role of Gender

The college impact model states that students’ background characteristics (e.g., gender) may make a difference in students’ college experiences as well as their learning outcomes. From a sociocultural perspective of learning, learning outcomes may vary depending on students’ background characteristics because of the role these characteristics (e.g., gender) play in shaping students’ learning experiences, values, and ultimately their engagement in the learning process [55]. Thus, male and female students in engineering programs with the same institutional support may have different learning outcomes because they have different understandings of the emphasis in the curriculum and different levels of engagement with the curriculum. A smaller percentage of women work in engineering than in other scientific and technical majors. Negative stereotypes of women’s engineering and math abilities have become a barrier to female academic achievement [56]. Knight et al. [57] surveyed students from 121 programs at 31 institutions and concluded that differential curricular emphasis, instructional practices, and student perceptions of climate were associated with gender differences in engineering disciplines. Kamphorst et al. [58] investigated male and female undergraduate engineering students at five Dutch universities and concluded that there were differences between male and female students in terms of background characteristics, engagement factors and academic success, and that there were gender differences in the relationship between background characteristics, engagement factors and academic achievement.

For sustainability learning outcomes, Olsson and Gericke [59] found that there is a gender gap in students’ sustainability awareness. Swift et al. [60] surveyed 228 junior and senior civil, environmental, and mechanical engineering students in order to explore what differences existed between men’s and women’s attitudes toward sustainability in upper-level engineering courses. The results of the study revealed that female engineers had different expectations of sustainability-related career outcomes. Synthesizing the above established studies, this study argues that sustainable development literacy, a key learning outcome for engineering undergraduates, may differ by student gender, particularly in terms of different college experiences and engagement in sustainable development learning processes. In the engineering education and sustainable development education literature, gender has emerged as an important factor influencing student achievement of learning outcomes, and studies have been conducted on gender as the moderator of the relationship between environment attitude and undergraduates’ behavior [61]. Therefore, this study proposes the following hypothesis:

**H3.**  *Gender significantly moderates the relationship between institutional support, student engagement, and sustainable development literacy*.

## 3. Materials and Methods

### 3.1. Sample and Data Collection

To test the questionnaire’s quality, 158 pilot questionnaires were distributed to fourth-year engineering undergraduates in Beijing before we formally issued the questionnaires. According to the analysis of the collected data, the design of some constructs and some ambiguous questions were revised. The formal questionnaires were randomly distributed among fourth-year engineering undergraduates from colleges and universities nationwide. Respondents of the survey were mainly from traditional engineering majors such as materials engineering, mechanical engineering, chemical engineering, and civil engineering. A total of 1194 questionnaires were collected via the internet, excluding invalid questionnaires such as those with too short a response time (the judgment criterion is that questionnaires with less than 60 s are invalid) and those with regular responses (the judgment criterion is that questionnaires with almost no difference in the options answered are invalid). Finally, 1070 valid questionnaires were collected, and the effective rate of the collected questionnaires is 89.6%. Among the participants, there were 532 female fourth-year engineering undergraduates, accounting for 49.7% of the total sample population, and 538 male fourth-year engineering undergraduates, accounting for 50.3% of the total sample population.

### 3.2. Tools for Measurement and Analysis of Variables

The questionnaire used in this study consists of two parts. The first part is about the undergraduates’ demographic information, including gender, birthplace, education, parents’ engineering background, family income, school, and class. The second part is about factors influencing the sustainability literacy of the engineering undergraduates. The questionnaire includes 31 questions to measure variables. The tools for measurement were designed on the basis of the college impact model and were revised according to the research hypotheses and expert suggestions.

The detailed statistical analysis of the subjects is shown in Table 1; demographic data such as gender, place of birth, parents’ education level, parents’ engineering background, family income, university type and performance ranking were investigated. Female respondents were slightly more numerous than males (538, 50.3%). Most respondents were born in the city (684, 63.9%). Most respondents’ parents had college degrees (445, 41.6%). About half of respondents’ parents had no engineering background (542, 50.7%). Respondents’ family income of 80,000–150,000 accounted for the largest share (358, 33.5%). In academic performance, the respondents who ranked in the top 11–25% accounted for the largest share (376, 35.1%). The majority of respondents are enrolled in “Double First-Class” universities in China (602, 56.3%). “Double First-Class” is another national strategy after the “211 Project” and “985 Project” in China; a total of 147 universities were evaluated as “Double First-Class” universities in 2022.

The scale of sustainable development literacy consists of five questions that refer to *Education for Sustainable Development Goals: Learning Objectives* published by UNESCO in 2017 [21], which classify sustainable development literacy into three domains: cognitive, socio-emotional, and behavioral. Considering the content of engineering education, the question items integrate the provisions of the Washington Accord [14] and ABET [15] for sustainability outcomes for engineering undergraduates. A question such as “I can accurately identify sustainability issues in engineering projects and analyze the causes of the problems” is required to be presented. Each item is scored on a 7-point Likert scale, with “strongly disagree” being assigned a score of 1, “neutral” a score of 4, and “strongly agree” a score of 7.

This study divided institutional support into three areas: supportive environment, sustainability curriculum, and instructional practice. Based on Hopkinson’s interpretation of the campus curriculum [39], this study compiled the question items of the supportive environment dimension. The supportive environment scale consists of six questions, referring to the institutional environment, resource, and technical environment. To measure sustainability curriculum and instructional practice, this study used the survey instrument developed by Lattuca et al. [62], while modifying the question items to be applicable to engineering education. The sustainability curriculum scale consists of five questions regarding students’ sustainable development literacy addressed in the curriculum and content. The instructional practice scale consists of five questions about teaching and learning around sustainable development literacy in the classroom. Each item is scored on a 7-point Likert scale, with “strongly disagree” being assigned a score of 1, “neutral” a score of 4, and “strongly agree” a score of 7.

To measure the extent and type of student engagement, this study adapted a survey instrument developed by the National Survey of Student Engagement (NSSE) [63] to categorize student engagement into two areas: classroom experience and extracurricular engagement. For the classroom experience variable, consisting of six questions, students were asked to respond to whether they actively participated in classroom discussions and teaching activities in the courses they took at the university level, such as “I actively participate in class discussions in the sustainability curriculum”. The extracurricular involvement variable consists of four questions that require students to answer questions about their participation in public service activities, lectures, social practices, etc., related to sustainable development in their school or department, student organizations or clubs, or off-campus organizations. Each item is scored on a 7-point Likert scale, with “strongly disagree” being assigned a score of 1, “neutral” a score of 4, and “strongly agree” a score of 7.

The study used SPSS 25.0 and AMOS 24.0 (IBM Corp., Armonk, NY, USA) statistical software for reliability analysis, descriptive statistics, and correlation analysis. AMOS 24.0 software was used to construct a structural model of the relationship between the study variables, and measurement model tests to determine the fit and reliability of the factor structure for testing the paths between the study variables. Further, with the help of AMOS 24.0 software, the mediating role of student engagement and the moderating effect of gender were tested using the Bootstrap method.

## 4. Results

### 4.1. Descriptive Analysis

As can be seen from Table 2, the means ranged from 5.141 to 5.541, the standard deviations ranged from 1.510 to 1.623, the skewness values ranged from −0.758 to −0.278, and the kurtosis values ranged from −1.028 to −0.551, which met the criteria of absolute value of skewness of less than 2 and absolute value of kurtosis of less than 7 proposed by Kline [64], indicating that the data meet the normal distribution. As can be seen from Table 2, the mean of “EI1: I actively participate in extracurricular science and technology competitions, innovation and entrepreneurship training, etc.” is the largest at 5.541, and the mean of “SE1: Compared to other majors, my school attaches great importance to the development of my major discipline” is the smallest at 5.141, representing that the respondents agree most with EI1 and less with SE1.

### 4.2. Confirmatory Factor Analysis

This study assessed the measurement and structural model adopting the two-step approach of Structural Equation Modeling (SEM) proposed by Anderson and Gerbing [65]. The first step using Confirmatory Factor Analysis (CFA) examined the construct reliability and validity of the measurement model. The second step tested the path effects and their significance in the structural model. By using the maximum likelihood estimation (MLE) in terms of factor loadings, reliability of measurement, convergent validity, and discriminant validity, the measurement model was assessed.

Table 3 presents a summary of unstandardized factor loadings, standard errors, significance tests, standardized factor loadings, square multiple correlations, composite reliability, and average variance extracted (AVE). Fornell and Larcker [66] proposed three indexes for assessing convergent validity of the measurement items. There are (a) item reliability of each measure or square multiple correlations, (b) composite reliability of each construct, and (c) the average variance extracted. In a construct, composite reliability refers to the internal consistency of reliability of all indicators.

As Table 3 shows, in addition to the second-order confirmatory factor about ISIS and SESE, all standardized factor loadings of questions are from 0.685 to 0.849, falling into a reasonable range. This demonstrates that all questions have convergent validity. All the composite reliability values of the constructs, ranging from 0.848 to 0.913, exceed 0.7 recommended by Nunnally and Bernstein [67], indicating that all constructs have internal consistency. Lastly, all average variance extracted (AVE) values, ranging from 0.528 to 0.709, exceed 0.5 suggested by Hair et al. [68] and Fornell and Larcker [66], showing that all constructs have adequate convergent validity.

### 4.3. Item Parcel

From the reliability and validity analysis tables, it was found that the standardized factor loadings of ISIS and SESE to first-order constructs were close to 1 or greater than 1. The results of SEM analysis yielded offending estimates (error variance were insignificant or negative error variance), mainly due to the high correlation between sub-constructs [69]. Therefore, any one sub-construct can represent the original one and be used as the basis for measurement without compromising the results of the study. According to Hair et al. [68], it is recommended to use any of the sub-constructs as a surrogate variable for the subsequent analysis. In this study, IP and EI were used as surrogate variables for the analysis because of the high convergent validity of these two sub-constructs.

### 4.4. Measurement Model Analysis

#### 4.4.1. Convergent Validity

Table 4 illustrates a summary of unstandardized factor loadings, standardized factor loadings, standard errors, significance tests, square multiple correlations, composite reliability, and average variance extracted (AVE). The three indexes for assessing convergent validity of the measurement items proposed by Fornell and Larcker [66] are (a) item reliability of each measure or square multiple correlations, (b) composite reliability of each construct, and (c) the average variance extracted. Composite reliability refers to the internal consistency of reliability of all indicators in a construct.

As Table 4 shows, all standardized factor loadings of questions are from 0.681 to 0.864, falling into a reasonable range. This demonstrates that all questions have convergent validity. All the composite reliability values of the constructs, ranging from 0.848 to 0.907, exceed 0.7 recommended by Nunnally and Bernstein [67], indicating that all constructs have internal consistency. Lastly, all average variance extracted (AVE) values, ranging from 0.528 to 0.709, exceed 0.5 suggested by Hair, Anderson, Tatham, and Black [68] and Fornell and Larcker [66], showing that all constructs have adequate convergent validity.

#### 4.4.2. Discriminant Validity

Comparing the square root of the average variance extracted (AVE) of a given construct with the correlations between the construct and the other constructs is the discriminant validity [66]. The indicators are more closely related to the construct than others if the square root of the AVE of a construct is greater than the off-diagonal elements in the corresponding rows and columns.

Table 5 shows that most of the constructs have a root mean square of AVE larger than those associated with other constructs, and only SDL is slightly smaller than IS, as the difference is less than 0.1, indicating that the difference is negligible [70]. Therefore, the discriminant validity of this study is acceptable.

### 4.5. Structural Model Analysis

#### 4.5.1. Model Goodness-of-Fit Test and Path Coefficients Results

This study performed structural model testing to examine the hypothesized relationships of the proposed model with the maximum likelihood method. Model fit indicators determine whether the sample data fit the structural equation model proposed. A variety of standards were recommended by Kline [71] and Schumacker and Lomax [72] to determine the model fit of a structural model. One hundred and ninety-four Confirmatory Factor Analysis (CFA) studies printed in the American Psychological Association journals from 1998 to 2006 were reviewed and compared by Jackson, Gillaspy Jr., and Purc-Stephenson [73] to create model fit report guidelines. They are χ^2^, DF, χ^2^/DF ratio, GFI, AGFI, RMSEA, SRMR, CFI, TLI (NNFI), etc.

Table 6 presents several model fit indicators as well as the recommended thresholds. Except for χ^2^, all model fit indicators exceed the recommended levels [72]. Because χ^2^ is sensitive to a large sample, the ratio of χ^2^ to its degree of freedom was computed, and the ideal ratio should be below three for a good model fit. Hu and Bentler [74] suggested that instead of evaluating each index independently, more strict combination rules should be applied to model fit indices to control type I errors.

The model fit indicators, as shown in Table 6, satisfy both the independent level of recommended fits and the combination rule. Thus, it has been proven that the proposed model has a good fit.

In Table 7, the results of path coefficients are given: (IS) (b = 0.478, *p* < 0.001) and (SE) (b = 0.280, *p* < 0.001) significantly impact (SDL); (IS) (b = 0.809, *p* < 0.001) significantly impact (SE).

**Figure 1:**
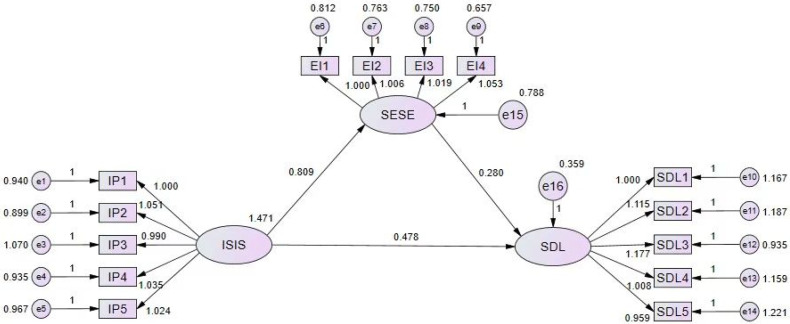
Research statistical model.

The results support the research question regarding the validity of the research model shown in Figure 1. A total of 68.8% of SDL can be explained by (IS) and (SE) constructs, and 55% of SE can be explained by (IS) constructs.

#### 4.5.2. Analysis of Mediation Effects

In a research model, Me is a mediator if the independent variable (X) affects the dependent variable (Y) through (Me). Because the mediator is closer to the outcome variable than the predictor variable, the mediator becomes a causal or endogenous variable. When the independent variable affects the dependent variable through the mediator, it is called a mediation effect. The indirect effect of the mediator can be examined by several methods such as Baron and Kenny’s approach (B-K method), product of coefficients, and bootstrapping mediation analysis.

In empirical studies, using bootstrapping mediation analysis is better than the B-K method or product of coefficient when evaluating indirect/mediation effects [75,76]. Because the assumption of normalized distribution of indirect effects can be ignored in the analysis, using bootstrapping mediation analysis has an advantage over the other two methods.

When bootstrapping, the product coefficient of a and b is estimated for each sampling with replacement. The distribution of the product of a and b derives standard errors and confidential intervals. It is recommended to set the number of resampling iterations to 5000, or at least 1000 [77]. Because bootstrapping mediation analysis can provide confidential intervals to examine the indirect effects, it is better than the other mediation testing methods. One of the preferable bootstrapping mediation analysis methods is bias corrected bootstrapping [76,78].

As shown in Table 8, the total effect IS→SDL, *p* < 0.05, bias-corrected confidence interval (CI) does not include 0 (CI of IS→SDL = [0.649 0.763]). The existence of total effect was supported. The total indirect effect IS→SE→SDL, *p* < 0.05, bias-corrected confidence interval (CI) does not include 0 (CI of IS→SE→SDL = [0.170 0.288]). The existence of total indirect effect was supported.

#### 4.5.3. The Path Coefficients Difference between Gender

Gender is a dichotomous variable, and Amos used multigroup analysis to compare the difference in the regression coefficients between gender. The premise of comparing the difference is that the factor loadings should remain equal, indicating that males and females have the same perception of the questionnaire [79]. Next, the coefficients were compared and if the difference was significant, there was a moderating effect, and if not, there was no moderating effect.

The results of the analysis are presented in Table 9 There was no difference in measurement weights (χ^2^_11,0.95_ = 3.388 < 19.675) and no significant difference in the structural path coefficients (χ^2^_23,0.95_ = 3.204 < 7.815). Therefore, there was no moderating effect for gender.

## 5. Discussion

### 5.1. The Impact of School Support on the Sustainable Development Literacy of Engineering Undergraduates

This empirical study found that institutional support has a significant, positive, direct impact on the sustainable development literacy of engineering undergraduates. Specifically, if colleges and universities provide comprehensive and strong environmental support for undergraduate engineering education and create a good curriculum experience for students, students’ sustainable development literacy will be significantly improved. As mentioned earlier, according to the college impact model and existing research on sustainable development literacy [39], a supportive campus environment is a key factor in student learning outcomes, and shaping a sustainable campus environment can help shape students’ sustainable development literacy in a subtle way. Sustainable learning environments, such as eco-schools or green campuses, allow educators and learners to integrate sustainability principles into their daily practices and facilitate capacity building, competency development, and value education in a comprehensive manner.

Consistent with the findings of existing studies [40,41,42], a sustainable curriculum and instructional practices also directly and positively affect the sustainable development literacy of engineering undergraduates. Formal curriculum is one of the most important ways for engineering undergraduates to learn professional knowledge. By integrating sustainability topics into the curriculum, engineering undergraduate students can be systematically developed in sustainable development literacy. Appropriate instructional practices such as project-based learning and problem-based learning can be effective in education for sustainable development [45,46]. Instruction includes, on the one hand, transferring information about sustainability knowledge and skills to students so that they can acquire and understand that knowledge, and on the other hand, learning and developing sustainability learning outcomes in greater depth.

### 5.2. The Mediating Role of Student Engagement

This study further found that in addition to the presence of a significant direct positive effect, institutional support had an impact on students’ sustainable development literacy through the mediating role of student engagement. This is consistent with Pascarella’s theoretical model [32] and existing empirical studies [51,52], where student learning outcomes depend heavily on the quality of individual learning efforts and the level of engagement in academic and extracurricular activities. Whether students have clear learning goals and strong motivation, whether they adopt appropriate learning methods, and whether they actively participate and engage in courses, programs, and activities that benefit their own learning and development are directly related to their ultimate academic achievement and personal development. This suggests that the active academic engagement of students as dynamic subjects is an important bridge between external educational resources and individual sustainable development literacy. These findings suggest that current curriculum content and instructional practices can, to some extent, stimulate students’ interest in academic engagement, enhance their motivation to participate academically, and ultimately influence the acquisition of sustainable development literacy. Education for sustainable development advocates that learning to learn is more important than learning to know, learning to innovate is more important than learning to inherit, and students’ self-development is more important than their development being passively shaped.

### 5.3. The Moderating Effect of Gender

This study found that there is no significant moderating effect of gender on the relationship between sustainable development literacy and student engagement, which is not consistent with the conclusions of some previous studies. Previous studies [56] argued that gender varies significantly in the field of engineering education, and that men’s psychological and physiological advantages are more conducive to sustainable professional knowledge learning.

The results of this study differ from previous studies [59,60], possibly due to sampling limitations that resulted in no significant difference in sustainable development literacy between male and female students who participated in this research study, and cannot be extrapolated to the entire group of engineering undergraduates. Another reason may be that females and males have the same expectation of sustainable learning outcomes and are able to actively engage in the classroom process to improve their own sustainable development literacy by giving full play to their own initiative. It has been confirmed that female engineering students have the same academic integration, active learning satisfaction, and academic knowledge skills satisfaction as male engineering students, and slightly higher social integration. Female engineering students spend even more time on independent study than males [80]. In addition to biological factors, this study concluded that females’ self-motivation level, achievement goals, and internal motivation are the same as those of male students. The different gender role tendencies of males and females do not affect their competencies of sustainable development literacy, and boys and girls can be cultivated equally to acquire knowledge to promote their own sustainable development.

## 6. Implications for Practice

Based on the theory of the college impact model, this study constructed a model of the factors influencing the sustainable development literacy of engineering undergraduates, while extending the scope of the college impact model and verifying the applicability of the college impact model to the study of sustainable development learning outcomes. Although institutional support is considered a key educational factor in developing students’ sustainable development literacy, there is limited quantitative evidence on the relationship between institutional support (i.e., supportive environment, sustainability curriculum, instructional practice) and sustainable development literacy among engineering undergraduates. This study verified the significant positive effect of institutional support on the sustainable development literacy of undergraduate engineering students. The findings of this study have practical implications for sustainability in engineering education, and higher education institutions can adopt curriculum revision, with the introduction of new courses and engaged pedagogy to incorporate sustainability goals into their teaching areas.

Furthermore, the college impact model states that institutional support not only affects student learning outcomes directly, but also indirectly by influencing student engagement. Therefore, this study used student engagement as a mediating variable between institutional support and engineering undergraduate students’ sustainable development literacy, which also indirectly influenced student engagement. The study found that student engagement partially mediated the relationship between institutional support and sustainable development literacy, suggesting that the active academic engagement of students as dynamic subjects is an important bridge between external educational resources and individual sustainable development literacy. Therefore, it is important to increase the motivation of engineering undergraduates to actively engage in sustainable development activities in future engineering education. Expanding student engagement requires the establishment of student-centered teaching practices. It is necessary to improve the participation of engineering undergraduates by innovating education and teaching methods and constructing more flexible teaching modes. To further cultivate their sustainable development literacy and ability, in the teaching process of sustainable higher engineering education, all the teachers should advocate the learner-centered teaching method and highlight the central position of engineering undergraduates.

Additionally, this study incorporated gender as a moderating variable in the college impact model, thus providing a more comprehensive view of the mechanisms of institutional support and student engagement on sustainability literacy in the context of engineering education. Based on the empirical analysis of this study, women and men have the same expectations for learning sustainable professional knowledge, and can actively participate in the process of classroom teaching by fully exerting their own subjective initiative, thus improving the effect of knowledge learning. This study helps promote the diversification of engineering education students. Promoting diversity and pluralism in the engineering education student body is also an important sustainability issue in its own right, and engineering education has long been committed to increasing the participation of people, including women and minorities, in engineering education and engineering careers because the participation of diverse populations brings new ideas and ways of thinking to the development of the engineering field.

## 7. Conclusions

The study examined the factors influencing undergraduate students’ sustainable development literacy. Based on the college impact model, a questionnaire survey was conducted among 1070 randomly selected fourth-year engineering undergraduate students at Chinese universities to explore the impact of institutional support on undergraduate students’ sustainable development literacy, and the structural model of sustainable development literacy was effectively tested. The results show that the direct effect of institutional support, sustainability curriculum, and instructional practice is significant on sustainable development literacy among engineering undergraduates. Student engagement significantly mediates institutional support and sustainable development literacy. Furthermore, gender significantly moderated the relationship between student engagement and sustainable development literacy, and males are significantly higher than females in this pathway relationship. Based on the empirical analysis and practical experience, it is suggested to cultivate engineering undergraduates with a high level of sustainability development literacy.

The study has some enlightenments for educators and administrators to integrate sustainable development literacy into higher engineering education, but there are still some limitations. First, the variables of the study are mainly based on the college impact model; however, in educational practice, many other relevant influencing factors should be involved, such as learning motivation, self-efficacy, peer effects, etc., for a more in-depth explanation. The influence of these factors on students’ sustainable development literacy needs to be further analyzed. Second, the study only surveyed undergraduates in engineering majors. However, sustainable development literacy is necessary for all students, and the sample size should be enriched to test influencing factors such as interdisciplinary vision and disciplinary variation in future study.

## Figures and Tables

**Table 1:** Results of statistical analysis of subjects (n = 1070).

Variable	Categories	Frequency	Valid Percent	Cum Percent
Gender	Male	532	49.7	49.7
	Female	538	50.3	100.0
	Total	100.0	100.0	
Place of birth	Urban	684	63.9	63.9
	Rural	386	36.1	100.0
	Total	1070	100.0	
Parents’ education level	Junior high school and below	164	15.3	15.3
	Senior high school	270	25.2	40.6
	Higher vocational institution	180	16.8	57.4
	College and university	445	41.6	99.0
	Postgraduate and above	11	1.0	100.0
	Total	1070	100.0	
Parents’ engineering background	Yes	528	49.3	49.3
	No	542	50.7	100.0
	Total	1070	100.0	
Family income	Below 30,000 RMB	44	4.1	4.1
	30,001-80,000 RMB	217	20.3	24.4
	80,001-150,000 RMB	358	33.5	57.9
	150,001-300,000 RMB	327	30.6	88.4
	Beyond 300,000 RMB	124	11.6	100.0
	Total	1070	100.0	
“Double First-Class” universities	Yes	602	56.3	56.3
	No	468	43.7	100.0
	Total	1070	100.0	
Performance ranking	Top 10%	284	26.5	26.5
	11%~25%	376	35.1	61.7
	26%~50%	279	26.1	87.8
	51%~75%	90	8.4	96.2
	Last 25%	15	1.4	97.6
	No idea	26	2.4	100.0
	Total	1070	100.0	

**Table 2:** Descriptive analysis.

Item	N	Basic Statistics					Normality Check	
		Min	Max	Mean	S.D.	Range/S.D.	Skewness	Kurtosis
CE1	1070	1.00	7.00	5.457	1.586	3.783	−0.611	−0.707
CE2	1070	1.00	7.00	5.306	1.612	3.722	−0.452	−0.944
CE3	1070	1.00	7.00	5.250	1.623	3.697	−0.413	−1.028
CE4	1070	1.00	7.00	5.421	1.601	3.748	−0.624	−0.753
CE5	1070	1.00	7.00	5.524	1.599	3.752	−0.742	−0.596
CE6	1070	1.00	7.00	5.479	1.587	3.781	−0.666	−0.649
EI1	1070	1.00	7.00	5.541	1.601	3.748	−0.758	−0.571
EI2	1070	1.00	7.00	5.492	1.593	3.766	−0.720	−0.584
EI3	1070	1.00	7.00	5.437	1.603	3.743	−0.597	−0.781
EI4	1070	1.00	7.00	5.369	1.613	3.720	−0.577	−0.816
IP1	1070	1.00	7.00	5.418	1.554	3.861	−0.584	−0.702
IP2	1070	1.00	7.00	5.339	1.589	3.776	−0.497	−0.825
IP3	1070	1.00	7.00	5.389	1.586	3.783	−0.559	−0.745
IP4	1070	1.00	7.00	5.343	1.585	3.785	−0.497	−0.816
IP5	1070	1.00	7.00	5.376	1.585	3.785	−0.494	−0.851
SC1	1070	1.00	7.00	5.394	1.568	3.827	−0.544	−0.746
SC2	1070	1.00	7.00	5.366	1.578	3.802	−0.537	−0.754
SC3	1070	1.00	7.00	5.309	1.602	3.745	−0.444	−0.936
SC4	1070	1.00	7.00	5.379	1.576	3.807	−0.524	−0.794
SC5	1070	1.00	7.00	5.326	1.577	3.805	−0.447	−0.891
SDL1	1070	1.00	7.00	5.257	1.523	3.940	−0.408	−0.722
SDL2	1070	1.00	7.00	5.167	1.619	3.706	−0.434	−0.776
SDL3	1070	1.00	7.00	5.198	1.592	3.769	−0.416	−0.736
SDL4	1070	1.00	7.00	5.246	1.527	3.929	−0.460	−0.623
SDL5	1070	1.00	7.00	5.287	1.510	3.974	−0.473	−0.551
SE1	1070	1.00	7.00	5.141	1.524	3.937	−0.278	−0.860
SE2	1070	1.00	7.00	5.225	1.608	3.731	−0.337	−0.988
SE3	1070	1.00	7.00	5.362	1.576	3.807	−0.502	−0.809
SE4	1070	1.00	7.00	5.341	1.580	3.797	−0.465	−0.844
SE5	1070	1.00	7.00	5.430	1.562	3.841	−0.577	−0.738
SE6	1070	1.00	7.00	5.371	1.608	3.731	−0.484	−0.916

CE: classroom experience; EI: extracurricular involvement; IP: instructional practice; SC: sustainability curriculum; SDL: sustainable development literacy; SE: supportive environment.

**Table 3:** Reliability and convergence validity analysis.

Construct	Item	Significant Test of Parameter Estimation				Item Reliability		Composite Reliability	Convergence Validity
		Unstd.	S.E.	Unstd./S.E.	*p*-Value	STD.	SMC	CR	AVE
CE	CE1	1.000				0.787	0.619	0.913	0.637
	CE2	1.008	0.035	28.608	0.000	0.780	0.608		
	CE3	1.016	0.036	28.585	0.000	0.781	0.610		
	CE4	1.030	0.035	29.420	0.000	0.803	0.645		
	CE5	1.051	0.035	30.251	0.000	0.821	0.674		
	CE6	1.036	0.035	30.008	0.000	0.815	0.664		
EI	EI1	1.000				0.832	0.692	0.907	0.709
	EI2	1.008	0.029	34.288	0.000	0.844	0.712		
	EI3	1.014	0.030	34.221	0.000	0.843	0.711		
	EI4	1.028	0.029	34.904	0.000	0.849	0.721		
IP	IP1	1.000				0.770	0.593	0.888	0.613
	IP2	1.034	0.037	27.599	0.000	0.779	0.607		
	IP3	1.011	0.038	26.854	0.000	0.763	0.582		
	IP4	1.071	0.037	28.851	0.000	0.808	0.653		
	IP5	1.052	0.037	28.146	0.000	0.794	0.630		
SC	SC1	1.000				0.747	0.558	0.871	0.575
	SC2	1.017	0.039	25.921	0.000	0.754	0.569		
	SC3	1.026	0.040	25.743	0.000	0.750	0.562		
	SC4	1.019	0.039	26.043	0.000	0.757	0.573		
	SC5	1.055	0.039	26.978	0.000	0.783	0.613		
SDL	SDL1	1.000				0.701	0.491	0.848	0.528
	SDL2	1.125	0.051	22.216	0.000	0.741	0.549		
	SDL3	1.184	0.050	23.568	0.000	0.794	0.630		
	SDL4	1.013	0.048	21.280	0.000	0.708	0.501		
	SDL5	0.969	0.047	20.417	0.000	0.685	0.469		
SE	SE1	1.000				0.689	0.475	0.879	0.549
	SE2	1.135	0.050	22.902	0.000	0.741	0.549		
	SE3	1.147	0.049	23.354	0.000	0.764	0.584		
	SE4	1.131	0.049	22.982	0.000	0.751	0.564		
	SE5	1.138	0.049	23.379	0.000	0.765	0.585		
	SE6	1.120	0.050	22.391	0.000	0.731	0.534		
ISIS	SE	1.000				0.972	0.945	0.997	0.990
	SC	1.170	0.051	22.865	0.000	1.019	1.038		
	IP	1.166	0.050	23.134	0.000	0.994	0.988		
SESE	CE	1.000				1.012	1.024	1.000	1.001
	EI	1.044	0.036	29.091	0.000	0.989	0.978		

CE: classroom experience; EI: extracurricular involvement; IP: instructional practice; SC: sustainability curriculum; SDL: sustainable development literacy; SE: supportive environment; ISIS: institutional support; SESE: student engagement.

**Table 4:** Results for the measurement model.

Construct	Item	Significance of Estimated Parameters				Item Reliability		Construct Reliability	Convergence Validity
		Unstd.	S.E.	Unstd./S.E.	*p*-Value	Std.	SMC	CR	AVE
SDL	SDL1	1.000				0.705	0.497	0.848	0.528
	SDL2	1.115	0.050	22.215	0.000	0.739	0.546		
	SDL3	1.177	0.050	23.609	0.000	0.794	0.630		
	SDL4	1.008	0.047	21.353	0.000	0.709	0.503		
	SDL5	0.959	0.047	20.369	0.000	0.681	0.464		
ISIS	IP1	1.000				0.781	0.610	0.888	0.614
	IP2	1.051	0.038	27.783	0.000	0.802	0.643		
	IP3	0.990	0.038	25.917	0.000	0.758	0.575		
	IP4	1.035	0.037	27.602	0.000	0.792	0.627		
	IP5	1.024	0.038	26.875	0.000	0.784	0.615		
SESE	EI1	1.000				0.826	0.682	0.907	0.709
	EI2	1.006	0.032	31.855	0.000	0.836	0.699		
	EI3	1.019	0.032	32.154	0.000	0.841	0.707		
	EI4	1.053	0.031	34.280	0.000	0.864	0.746		

Unstd.: unstandardized factor loadings; Std: standardized factor loadings; SMC: square multiple correlations; CR: composite reliability; AVE: average variance extracted. IS: institutional support; SE: student engagement; SDL: sustainable development literacy.

**Table 5:** Discriminant validity for the measurement model.

	AVE	SDL	ISIS	SESE
SDL	0.528	**0.727**		
ISIS	0.614	0.797	**0.784**	
SESE	0.709	0.746	0.741	**0.842**

The items on the diagonal in bold represent the square roots of the AVE; off-diagonal elements are the correlation estimates. IS: institutional support; SE: student engagement; SDL: sustainable development literacy.

**Table 6:** Model fit.

Model Fit	Criteria	Model Fit of Research Model
MLχ^2^	The small the better	179.679
DF	The large the better	74.000
Normed Chi-sqr (χ^2^/DF)	1 < χ^2^/DF < 3	2.428
RMSEA	<0.08	0.037
SRMR	<0.08	0.019
TLI (NNFI)	>0.9	0.986
CFI	>0.9	0.988
GFI	>0.9	0.98
AGFI	>0.9	0.976

**Table 7:** Regression coefficient.

DV	IV	Unstd	S.E.	Unstd./S.E.	*p*-Value	Std.	R^2^
SDL	IS	0.478	0.040	12.098	0.000	0.540	0.688
	SE	0.280	0.034	8.366	0.000	0.346	
SE	IS	0.809	0.038	21.563	0.000	0.741	0.550

IS: institutional support; SE: student engagement; SDL: sustainable development literacy.

**Table 8:** The analysis of indirect effects.

Effect	Point Estimate	Product of Coefficients			Bootstrap 1000 Times	
					Bias-Corrected 95%	
		S.E.	Z-Value	*p*-Value	Lower Bound	Upper Bound
Total effect						
IS→SDL	0.705	0.029	23.903	0.000	0.649	0.763
Total indirect effect						
IS→SE→SDL	0.227	0.029	7.711	0.000	0.170	0.288
Direct effect						
IS→SDL	0.478	0.037	12.815	0.000	0.406	0.555

Note: IS: institutional support; SE: student engagement; SDL: sustainable development literacy.

**Table 9:** Comparison regressing coefficients between gender.

Model	NPAR	χ^2^	DF	ΔDF	Δχ^2^	*p*
Unconstrained	62	256.147	148			
Measurement weights	51	259.535	159	11	3.388	0.985
Structural weights	48	262.739	162	3	3.204	0.361

NPAR: number of parameters.

## Data Availability

This data that support the findings of this study are available from the corresponding author upon reasonable request.
